# Controllable Patterning of Metallic Photonic Crystals for Waveguide–Plasmon Interaction

**DOI:** 10.3390/nano13040629

**Published:** 2023-02-05

**Authors:** Yuanhai Lin, Deqing Che, Wenjie Hao, Yifei Dong, Heng Guo, Junsheng Wang, Xinping Zhang

**Affiliations:** 1 Liaoning Key Laboratory of Marine Sensing and Intelligent Detection, Dalian Maritime University, Dalian 116026, China; 2 Information Science and Technology College, Dalian Maritime University, Dalian 116026, China; 3 Institute of Information Photonics Technology and College of Applied Sciences, Beijing University of Technology, Beijing 100124, China

**Keywords:** metallic photonic crystals, waveguide–plasmon polaritons, patterning, Fano coupling

## Abstract

Waveguide–plasmon polaritons sustained in metallic photonic crystal slabs show fascinating properties, such as narrow bandwidth and ultrafast dynamics crucial for biosensing, light emitting, and ultrafast switching. However, the patterning of metallic photonic crystals using electron beam lithography is challenging in terms of high efficiency, large area coverage, and cost control. This paper describes a controllable patterning technique for the fabrication of an Ag grating structure on an indium–tin oxide (ITO) slab that enables strong photon–plasmon interaction to obtain waveguide–plasmon polaritons. The Ag grating consisting of self-assembled silver nanoparticles (NPs) exhibits polarization-independent properties for the excitation of the hybrid waveguide–plasmon mode. The Ag NP grating can also be annealed at high temperature to form a continuous nanoline grating that supports the hybrid waveguide–plasmon mode only under transverse magnetic (TM) polarization. We tuned the morphology and the periodicity of the Ag grating through the concentration of silver salt and the photoresist template, respectively, to manipulate the strong coupling between the plasmon and the waveguide modes of different orders.

## 1. Introduction

Photon–plasmon interaction in metallic nanostructures allows flexible tailoring of their linear and nonlinear optical properties critical for various applications, such as enhanced spectroscopy [1], nanoantenna [2], and quantum plasmonics [3]. Waveguide–plasmon polaritons are quasiparticles formed by strong Fano coupling between photons and plasmons in metallic photonic crystals arranged on a dielectric slab [4,5]. The discrete state of the narrow optical waveguide mode in the slab and the continua state of the plasmon mode sustained by the metallic unit cells can destructively interfere when they are spectrally overlapped [6]. Consequently, almost complete suppression in light extinction can be obtained within a narrow bandwidth to achieve an electromagnetically induced transparency window [7,8]. In addition, the radiation characteristics of the waveguide–plasmon polaritons can be significantly modified by tuning the photon–plasmon coupling strength through the structural parameters. For example, the local optical density of states is reduced in the strong coupling regime, and the ultrafast dephasing time of the plasmon (~12 fs) is increased by more than a factor of two compared with the uncoupled system [9]. Lengthening the dephasing time of the particle plasmons directly leads to enhancing the local electric field [10], which facilitates the light–matter interaction process, such as the transverse magneto-optical Kerr effect [11], light emission [12,13], and photochemistry [14].

The coupling mechanism of the waveguide–plasmon polaritons enables a high degree of freedom to manipulate their optical response by structural geometries [15], such as the materials and thickness of the waveguide or the shapes and dimensions of the metallic unit cells. One-dimensional (1D) periodic gold nanowire arrays on an ITO waveguide can introduce strong photon–plasmon coupling with large Rabi splitting [4], which is suitable for photonic band gap engineering to manipulate the propagation properties of light [16,17,18]. Rearranging the periodic gold nanowires into a more complex superlattice can prolong the dephasing time of the waveguide–plasmon polaritons to produce the nonlinear effect of third-harmonic generation [10,19]. Besides the metallic photonic crystals, the dielectric layer can also be replaced by a magneto-optical thin film so that the waveguide–plasmon polaritons can significantly enhance the Faraday rotation effect [20]. Moreover, periodic metallic nanowires patterned on a semiconductor layer of TiO_2_ enable hot charge–carrier transfer from the metal to the semiconductor on the resonance of the hybrid waveguide–plasmon mode [14,21,22]. Two-dimensional (2D) metallic nanostructure arrays with tunable periodicities in the *x*- and *y*-axis directions can also be engineered on the dielectric layer to excite multi-modes of waveguide–plasmon polaritons [7,23,24]. Gold NP arrays on an ITO waveguide support two hybrid waveguide–plasmon resonances derived from the interaction between the plasmon polaritons and the transverse electric (TE) or transverse magnetic (TM) waveguide modes [8,25]. To enhance light emission, hexagonal Ag dot arrays on the ITO electrode were combined into the polymer light-emitting diodes to increase light extraction by Bragg scattering of waveguide modes [26]. Square arrays of rectangular Ag holes were designed on a uniform Bi:YIG film to enhance the magneto-optics through the interaction between the plasmon and waveguide modes [27]. Those metallic photonic crystal slabs have shown great potential in linear, nonlinear, and magneto-optics; however, the fabrication of the metallic structures relied on electron beam lithography, which offers limitations in terms of patterning areas and efficiency [28,29].

Here, we report a controllable fabrication technique to pattern a large-area metallic photonic crystal slab to introduce the Fano coupling scheme for excitation of the waveguide–plasmon polaritons. A 1D Ag grating structure on an ITO waveguide was fabricated using the photo-reduction method of Ag ions doped in a periodically modulated PVP polymer matrix. The grating structure composed of Ag NPs was fabricated after four-step patterning procedures and showed polarization-independent properties for the excitation of waveguide–plasmon polaritons. The Ag NP grating was then annealed at a high temperature to obtain continuous nanoline grating, which supports the waveguide–plasmon mode only under the TM polarization perpendicular to the grating direction. In addition, the photon–plasmon interaction was flexibly modified by altering the morphology and periodicity of the Ag grating through the concentration of the Ag salt and the grating template parameter, respectively.

## 2. Materials and Methods

**Figure 1:**
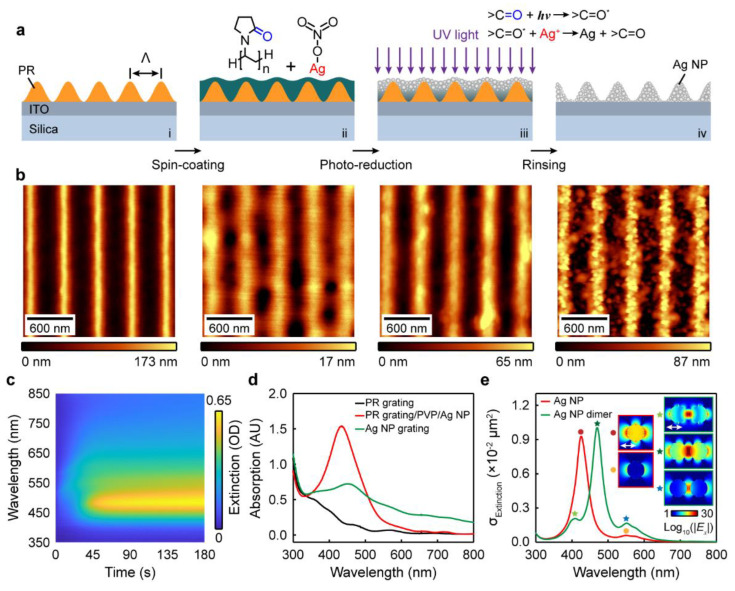
Patterning of Ag NP grating structure on an ITO waveguide. (**a**) Scheme for the fabrication process of the nanostructures. (**b**) AFM images of the grating structures in each fabrication steps. (**c**) Extinction spectra versus irradiation time of the sample measured during the photo-reduction process. (**d**) Absorption spectra of the PR grating in step i, the grating after photo-reduction in step iii, and the Ag NP grating in step iv. (**e**) Simulated extinction spectra of a single Ag NP and two coupled Ag NPs. Inset: electric field distributions of the plasmon resonances, white arrows denote the polarization direction of the incident light.

Figure 1a shows the patterning procedures of the Ag NP gratings using the method of the photo-reduction of silver ions [30,31]. Briefly, a photoresist (PR) grating template with a periodicity of 450 nm and an overall area larger than 0.785 cm^2^ (i.e., 10 mm in diameter) was fabricated using interference lithography on a glass substrate coated with a 180 nm-thick ITO layer (step i). The ITO thin layer was used as a planar waveguide to support the optical guided mode. Then, a mixed aqueous solution of silver nitrate (AgNO_3_) (Sigma-Aldrich Merck Ltd, Beijing, China) and poly (vinyl pyrrolidone) (PVP) (Sigma-Aldrich Merck Ltd, Beijing, China) was spin-coated onto the PR grating template at a speed of 2500 rpm for 30 s (step ii). The mixed aqueous solution was prepared by dissolving 0.4 g AgNO_3_ into 2 mL deionized (DI) water and 0.25 g PVP into 8 mL DI water, separately, before these two solutions were mixed together. Thus, the concentrations of the AgNO_3_ and the PVP were 3.8 and 2.3 wt. %, respectively. Next, the spin-coated sample was illuminated by a collimated 325 nm laser beam (Kimmon Koha Co., Ltd., Tokyo, Japan) with a power density of 39.8 mW/cm^2^ for 70 min (step iii). Under UV laser irradiation, the carbonyl group (>C=O) of the PVP absorbs the photon energies and produces an excited species (>C=O^*^). The photoexcited carbonyls reduce the silver ions (Ag^+^) to silver NPs within the patterned polymer film [30]. After the photo-reduction process, the sample was immersed in 50 mL ethanol (Sigma-Aldrich Merck Ltd, Beijing, China) and 50 mL acetone (Sigma-Aldrich Merck Ltd, Beijing, China) sequentially for 10 min and rinsed with DI water before drying at room temperature. The PVP polymer matrix and the PR grating were removed during the lift-off process, and the Ag NPs self-assembled to form a grating structure on the ITO-coated glass substrate (step iv).

To track the structural changes during the fabrication process, we measured the atomic force microscopy (AFM) (WITec Inc., Ulm, BaWü, Germany) images of the grating on each step, as shown in Figure 1b. The PR grating template with the periodicity of *Λ* = 450 nm has a groove depth of *d*_PR_ = 173 nm and a duty cycle of about 54%. After the spin-coating process, the PVP polymer matrix doped with Ag salt was filled into the PR grating grooves to form a shallow grating structure with a modulation depth of *d*_polymer_ = 17 nm. Then, the UV light irradiation induced nucleation and growth of the Ag NPs, which led to a slight collapse of the polymer matrix so that the photo-reduced grating had a larger groove depth of *d*_polymer-Ag NP_ = 65 nm. The Ag NPs self-assembled to form NP grating with a groove depth of *d*_Ag NP_ = 87 nm through Van der Waals force after the RP grating template and the PVP polymer matrix were lifted off.

In the waveguide–plasmon coupled system, the resonances of the localized surface plasmons (LSPs) are strongly correlated with the geometry and dimensions of the metallic nanostructures. Therefore, the structural parameters and arrangements of the Ag NPs in the gratings directly determine the coupling behaviors of the waveguide and plasmon modes. To characterize the LSPs, we first measured the extinction spectra of the pattern in real time during the photo-reduction process in step iii to monitor the growth of the Ag NPs, as shown in Figure 1c. In the measurement, a collimated white light was normally incident onto the irradiation area of the pattern, and the transmitted light was measured using a fiber spectrometer (Ocean optics, Inc., Dunedin, FL, USA). The optical extinction representing the sum of absorption and scattering was calculated using −log[*I*(*λ*)/*I*_0_(*λ*)] and has no unit [32,33,34,35], where *I*(*λ*) and *I*_0_(*λ*) are the transmission spectrum through the irradiation area and the ITO-coated glass substrate, respectively. In the measured extinction spectra, a broad peak of the LSPs with the central wavelength of *λ* ≈ 480 nm begins to appear at the irradiation time of *t*_irrad._ ≈ 30 min, denoting the formation of the Ag NPs. With increasing the irradiation time, this peak gradually increases in intensity due to the growth and increased numbers of the Ag NPs. When *t*_irrad._ ≥ 75 min, no significant changes of the spectra are observed, since the nucleation and growth of the Ag NPs were completed. The growth dynamics demonstrate that sufficient irradiation time (*t*_irrad._ > 30 min) is needed to ensure the excitation of the LSPs.

To verify that the extinction peak in Figure 1c derives from the LSPs of the Ag NPs, we compared the absorption spectra of the gratings obtained from the three fabrication steps: the PR grating (step i), the polymer matrix grating with photo-reduced Ag NPs (step iii), and the Ag NP grating (step iv), as shown in Figure 1d. We used the spectrophotometer of Agilent 8453 (Agilent Technologies, Inc., Palo Alto, CA, USA) to record the transmission of light passing through the sample and utilized the ChemStation software (B.04.03 Edition, Agilent Technologies, Inc., Melbourne, VIC, Australia) to obtain the absorption spectra. The PR grating has a progressively decreasing absorption band in the UV spectral region, which proves that the PR makes little contribution to the extinction peak. In contrast, a Lorenz-shaped peak shows up at *λ* = 432 nm in the absorption spectrum of the photo-reduced Ag NP grating, which confirms the resonances of the LSPs from the Ag NPs. Thus, the extinction peak at *λ* ≈ 480 nm is mainly due to the plasmon resonance of the Ag NPs instead of the absorption of the other materials. A discrepancy in peak position between the extinction and absorption spectra could come from the scattering part of the sample. After the rinsing process, the absorption of the Ag NP grating significantly decreases in intensity because of the loss of the Ag NPs. Moreover, the LSPs redshift to *λ* = 455 nm and broaden in spectral bandwidth.

To reveal the mechanism of the spectral redshift, we performed finite-difference time domain (FDTD) simulations to calculate the optical responses of a single Ag NP and an Ag NP dimer immersed in a PVP environment, as shown in Figure 1e. The single NP represents the photo-reduced Ag NPs separated from each other by the polymer matrix obtained in step iii, and the dimer characterizes the self-assembled Ag NPs with a 5 nm gap after the rinsing process in step iv. The red curve shows the extinction spectrum of the single Ag NP with a diameter of 25 nm, where a resonance peak is observed at *λ* = 426 nm (denoted by the dark red dot). The electric field distribution shows that this resonance corresponds to a quadrupolar plasmon, as shown in the inset of Figure 1e. A small peak (λ = 548 nm, indicated by the orange dot) also shows up at the right side of the quadrupolar mode, which can be assigned as a dipolar plasmon. When the Ag NPs were placed together to form a dimer system, the dominant peak redshifts from *λ* = 426 nm to *λ* = 470 nm because of strong near-field interaction between the two NPs. Numerical calculation verifies that this spectral feature corresponds to a hybrid quadrupolar plasmon (denoted by the dark cyan star). In addition, two shoulder peaks at *λ* = 406 nm (light green star) and 551 nm (navy blue star) originate from the resonances of hybrid hexapolar and quadrupolar LSPs, respectively. Therefore, the aggregation of the Ag NPs modifies the resonances of the Ag NPs and leads to a spectral redshift.

## 3. Results and Discussion

**Figure 2:**
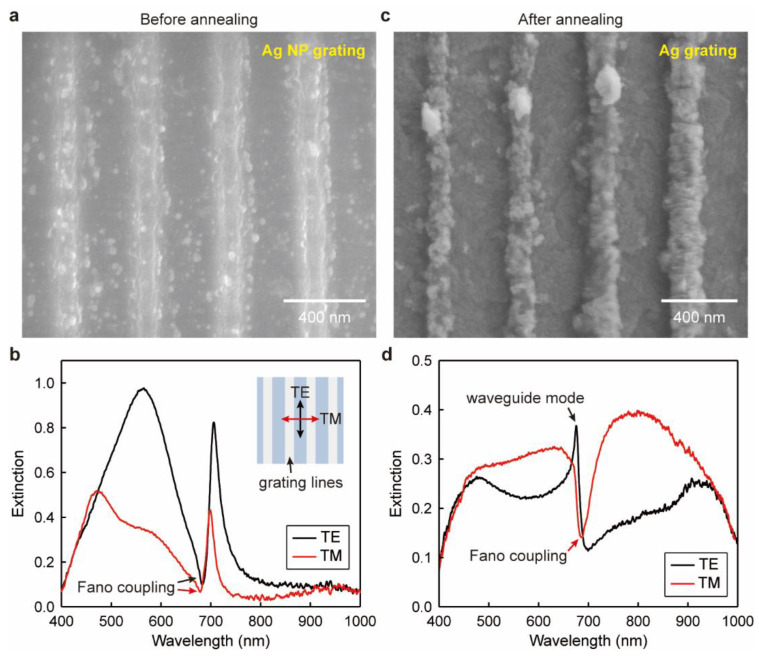
Ag NP grating slab structure modified by high-temperature annealing. (**a**) SEM image of the Ag NP grating and (**b**) the corresponding extinction spectra measured under the TE and TM polarizations. Inset: geometry of the optical measurement. (**c**) SEM image of the Ag grating and (**d**) the TE and TM extinction spectra.

Figure 2a depicts the scanning electron microscopy (SEM) (Hitachi High-tech, Gr., Tokyo, Japan) image of the Ag NP grating obtained after the rinsing process. Because the PVP polymer was dissolved into the ethanol and the PR into the acetone, the photo-reduced Ag NPs within the PR grooves fell onto the ITO substrate to form the ridges of the Ag NP grating. Thus, the grating lines shown in Figure 2a consist of the Ag NPs with a diameter of 25~30 nm. Note that the PVP polymer was not completely removed during the rinsing procedure; some Ag NPs are still buried in the polymer matrix. Besides this, we can also observe a small amount of Ag NPs left in the grooves of the Ag NP grating; those were initially produced on top of the PR grating ridges. The Ag NP grating on the planar ITO waveguide enables photon–plasmon interaction to excite the waveguide–plasmon polaritons. Thus, we measured the extinction spectra of the Ag NP grating under two orthogonal polarizations (i.e., TE and TM) that are parallel and perpendicular to the grating direction, as shown in Figure 2b. A broad peak over the spectral ranges of *λ* = 400~750 nm is observed under the TE polarization, which derives from the LSPs of the Ag NPs with wide ranges of diameters. Moreover, a narrow and asymmetric dip at *λ* = 681 nm emerges in the broad peak, corresponding to the Fano coupling between the LSPs and the waveguide mode. The waveguide mode propagating inside the ITO layer appears typically as a narrow extinction peak; however, it couples with the LSPs of the Ag NPs to form the waveguide–plasmon polaritons and manifests as an asymmetric extinction dip. Similarly, a Fano coupling spectral feature at *λ* = 678 nm also shows up at the right edge of a broad extinction band (*λ* = 400~726 nm) of the LSPs of the Ag NPs under the TM polarization. We noticed that two peaks could be distinguished at *λ* = 471 and 574 nm within the LSP spectral envelope, which can be attributed to the resonances of the quadrupole–quadrupole and dipole–dipole hybrid plasmons, respectively. Moreover, the intensity of the LSPs under the TM polarization is smaller than that under the TE case due to weaker NP interaction in the perpendicular direction of the grating. The Ag NP grating slab allows photon–plasmon interaction to obtain the waveguide–plasmon polaritons under both the TE and TM polarizations, which is crucial for applications in non-polarizing devices such as narrow-band filters and biosensors.

The polarization-independent properties for photon–plasmon interaction can also be modified by altering the structural morphology of the Ag grating. Thus, we annealed the sample in a Muffle furnace (Thermo Fisher Scientific, Inc., Waltham, MA, USA) at a temperature of 280 degrees to melt and fuse the Ag NPs into continuous nanolines. Figure 2c shows the SEM image of the grating structures consisting of uniform nanolines after the annealing process. Because the polymer residuals were sublimated during the annealing process, the imaged grating nanolines are clearer and more compact when compared to those in Figure 2a. Moreover, all the Ag NPs were fused into the continuous grating ridges; thus, no round-shaped NPs were observed on the grating lines and the grooves. Figure 2d shows the extinction spectra of the Ag grating slab under the TE and TM polarizations. Since the Ag grating does not support the LSPs under the TE polarization, the photon–plasmon interaction is quenched. Only a narrow peak of the waveguide mode with full width at half maximum of FWHM ≈ 18 nm is excited at *λ* = 680 nm. A broad background is also observed under this polarization, which can be ascribed to the scattering of light induced by the rough surface of the Ag grating. In comparison, a Fano resonance dip at *λ* = 682 nm emerges in a broad extinction band under the TM polarization. The broad band originates from the LSPs of the Ag grating lines as well as the scattering effect of the sharp structural features. Therefore, the Fano coupling between the LSPs and the waveguide mode is only achieved under the TM polarization after annealing the Ag NP grating structure.

**Figure 3:**
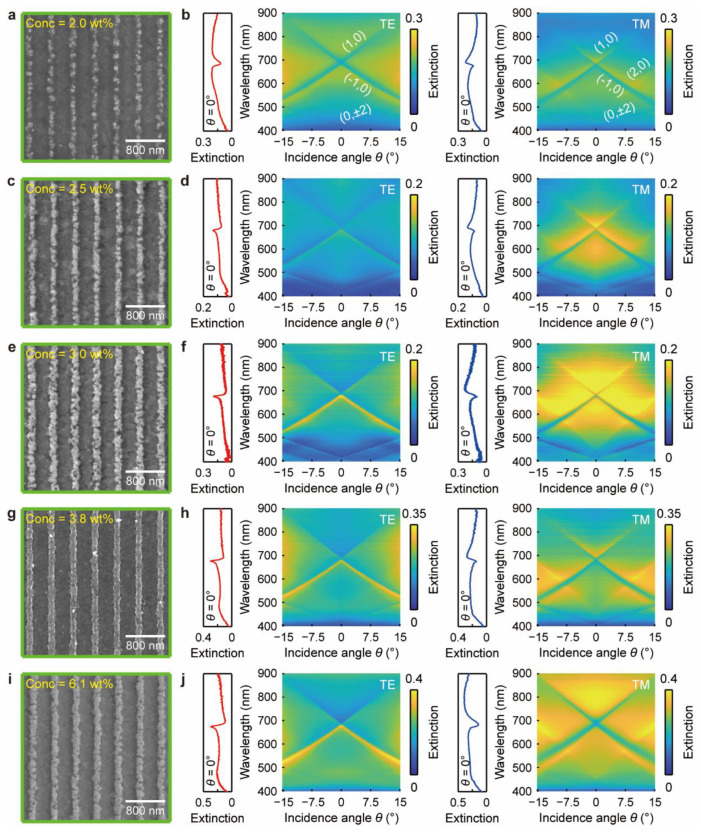
The morphologies and the optical responses of the Ag grating slabs tuned by the AgNO_3_ concentrations. (**a**–**j**) The SEM images and the corresponding angle-resolved TE and TM extinction spectra of the Ag grating slab structures fabricated using the AgNO_3_ concentrations of *Conc.*_AgNO3_ = 2.0, 2.5, 3.0, 3.8, and 6.1 wt. %.

To control the morphology of the Ag grating, we can also alter the concentration of the silver nitrate (*Conc.*_AgNO3_) to adjust the amount of the photo-reduced Ag NPs directly. The mixed aqueous solutions with different *Conc.*_AgNO3_ were prepared by only changing the quality of the AgNO_3_ dissolved in the DI water and maintaining that of the PVP. For example, we dissolved 0.21, 0.28, 0.32, 0.4, and 0.67 g AgNO_3_ into 2 mL DI water and 0.25 g PVP into 8 mL DI water to obtain the mixed aqueous solutions with *Conc.*_AgNO3_ = 2.0, 2.5, 3.0, 3.8, and 6.1 wt. %, respectively. Figure 3 shows the SEM images and the corresponding extinction spectra of the Ag grating slab structures fabricated using different AgNO_3_ concentrations. When *Conc.*_AgNO3_ = 2.0 wt. %, the amount of the Ag ions was insufficient to produce adequate NPs for forming continuous grating lines after annealing. Therefore, the grating lines are composed of isolated large-size Ag NPs and have a ridge width as narrow as 104 nm. The measured extinction spectra under the normal incidence of the TE and TM polarized lights show that Fano coupling between the photonic and plasmonic modes occurs at around *λ* = 680 nm to produce a narrow dip of the waveguide–plasmon polaritons, as shown by the red and blue curves in Figure 3b. With increasing the incidence angle, the coupled mode splits into two branches and follows the (±1, 0) diffraction modes, implying that the waveguide–plasmon polaritons maintain the same dispersion relations of the photonic modes. In addition, we also observed the (0, ±2) diffraction modes in the TE spectra and the (2, 0) and (0, ±2) diffraction modes in the TM spectra, which denotes that the Ag grating structure has a high diffraction efficiency in exciting the waveguide mode. We also noticed that the LSP bandwidth under the TE polarization (*λ*_LSP-TE_ = 450~880 nm) is much broader than that under the TM polarization (*λ*_LSP-TM_ = 450~740 nm), as shown by the yellow region in Figure 3b, which can be attributed to the broad NP size distributions along the grating direction.

When the concentration was increased to *Conc.*_AgNO3_ = 2.5 wt. %, the Ag grating lines have a larger width of 119 nm and become more continuous even though some fractures can be observed, as shown in Figure 3c. The measured extinction spectra under the normal incidence show that the LSPs were only excited under the TM polarization and coupled with the waveguide mode to generate a Fano resonance dip at *λ*_LSP-TM_ = 679 nm. In contrast, a narrow peak of the pure waveguide mode is observed in the TE extinction spectrum. The angle-resolved spectra further verify the polarization-dependent Fano coupling behaviors between the photonic and plasmonic modes. As the concentration was increased to *Conc.*_AgNO3_ = 3.0 wt. %, the Ag grating lines have the largest width of 138 nm and have more sharp and random structural features. Therefore, the LSPs excited by the TM polarized light have a broad resonance bandwidth of Δ*λ* ≈ 850−450 nm ≈ 400 nm, and thus couple with both the (±1, 0) waveguide modes. Most importantly, a well-defined sharp peak of the waveguide mode is obtained under the TE polarization because of the high diffraction efficiency of the Ag grating.

When we further increased the AgNO_3_ concentration to *Conc.*_AgNO3_ = 3.8 wt. %, a high-quality Ag grating structure with uniform and continuous nanolines was fabricated, as shown in Figure 3g. The grating nanolines have a smaller width of 125 nm than that obtained from the condition of *Conc.*_AgNO3_ = 3.0 wt. %, because all the photo-reduced Ag NPs were fully melted and fused to form more compact nanolines. Similarly, Fano coupling of the waveguide and plasmon modes is only obtained under the TM polarization. Because of the narrower bandwidth of the LPRs (Δ*λ* ≈ 750−460 nm ≈ 290 nm), the plasmon mode has a stronger coupling strength with the (−1, 0) waveguide mode than with the (1, 0) mode. When *Conc.*_AgNO3_ = 6.1 wt. %, the highly concentrated Ag ions in the mixed aqueous solution are extremely active and can be directly reduced to Ag NPs under background light. Most of the PVP agents and the Ag ions were consumed before spin-coating; hence, insufficient amounts of Ag NPs were photo-reduced under the illuminations of the UV laser. Consequently, we obtained an Ag grating structure consisting of fractured nanolines with a width of 145 nm. The measured angle-resolved extinction spectra show that the LSPs with a very broad spectral linewidth of Δ*λ* ≈ 900−460 nm ≈ 440 nm are excited under the TM polarization, and strongly couple with the (±1, 0) waveguide modes to generate the waveguide–plasmon polaritons. Therefore, we changed the AgNO_3_ concentrations to flexibly manipulate the Fano coupling of the photon–plasmon modes, which provides an efficient way to control the light–matter interaction.

**Figure 4:**
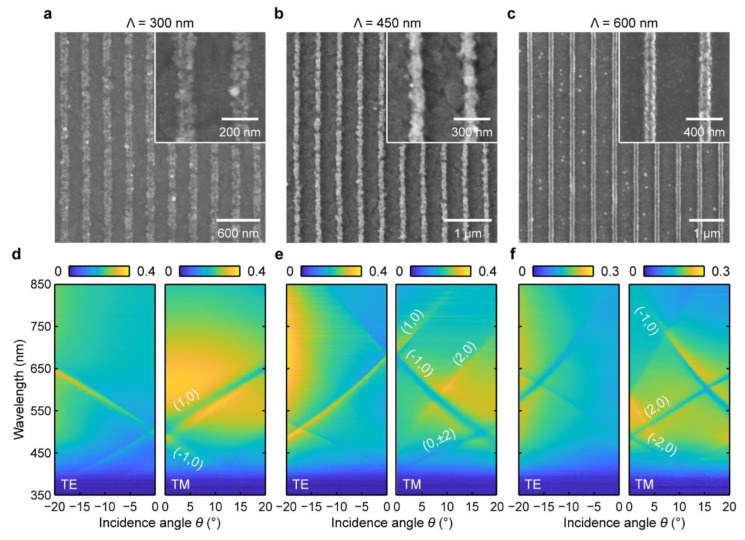
Tuning the waveguide–plasmon coupling behavior by the periodicity of the Ag grating. (**a**–**c**) SEM images of the Ag gratings with the periodicity of *Λ* = 300, 450, and 600 nm. (**d**–**f**) the corresponding angle-resolved extinction spectra of the Ag gratings under the TE and TM polarizations.

Besides the morphology of the nanolines, we also altered the periodicity of the Ag grating to modify the Fano coupling between the LSPs and the waveguide modes of different orders. Figure 4a–c show the SEM images of the Ag gratings with the periodicities of *Λ* = 300, 450, and 600 nm fabricated under the same condition of *Conc.*_AgNO3_ = 3.8 wt. % and *T*_ann._ = 280 ℃. With increasing the periodicity, the PR grating template has wider grooves to contain more materials to produce a larger number of Ag NPs. Thus, the fabricated Ag grating with a larger periodicity has more compact and uniform nanolines. The SEM images demonstrated that the Ag grating with the periodicity of *Λ* = 300, 450, and 600 nm has a ridge width of *d*_w_ = 93, 106, and 116 nm, respectively. Figure 4d–f depict the angle-resolved extinction spectra of the three Ag grating slab structures under the TE and TM polarizations. At *Λ* = 300 nm, the two polarized lights excited the first-order (±1, 0) waveguide modes at *λ* = 502 nm under the normal incidence. With increasing the incident angle from *θ* = 0° to −20°, the (−1, 0) and (1, 0) waveguide modes under the TE polarization correspondingly red- and blueshift to *λ* = 645 and 360 nm. Under the TM polarization, the (−1, 0) waveguide mode shows a similar spectral evolution to that in the TE case. However, the (1, 0) waveguide mode coupled with the plasmon mode to form the waveguide–plasmon polaritons because of the excitation of the LSPs at the resonance wavelength range of *λ* = 450~750 nm.

When the periodicity was increased to *Λ* = 450 nm, the first-order waveguide modes redshift to *λ* = 678 nm under the normal incidence, and the second-order waveguide modes appear at about *λ* = 410 nm. Under the TE polarization, the uncoupled (−1, 0) waveguide mode blueshifts to *λ* = 470 nm when the incidence angle increases to *θ* = −20°, and the degenerated (0, ±2) modes redshift to *λ* = 513 nm. A broad resonance at *λ* = 430~850 nm emerges in the spectra when the incidence angle is larger than *θ* = −14°, which can be ascribed to the LSPs excited by the TE component along the *z*-axis direction. Under the TM polarization, the LSPs were excited at the resonance wavelength of *λ* = 450~750 nm and coupled with the (−1, 0) waveguide mode to produce a Fano resonance dip evolved from *λ* = 676 to 470 nm. Moreover, the (1, 0), (2, 0), and (0, ±2) waveguide modes were also observed on the angle-resolved TM extinction spectra. As the periodicity was further increased to *Λ* = 600 nm, the first- and second-order waveguide modes redshift to *λ* = 485 and 860 nm at *θ* = 0°, respectively. The (2, 0) waveguide mode strongly couples with the LSPs as the incident angle increased from *θ* = 0° to 20° under the TM polarization, while the (−1, 0) mode starts to couple with the LSPs when *θ* = 10°. In contrast, the (−1, 0) (2, 0) waveguide modes also couple with the LSPs at a large incident angle (*θ* > 7°) under the TE polarization. Therefore, we realized the manipulation of the photon–plasmon interaction by varying the grating periodicities, which enables an efficient strategy to engineer the plasmonic device for applications in sensing, optical switching, and nonlinear optics.

## 4. Conclusions

In summary, we achieved strong Fano coupling between the plasmon and waveguide modes from an Ag grating structure on ITO-coated substrate patterned by the photo-reduction of Ag ions in a periodically modulated PVP polymer matrix. A uniform Ag NP grating as large as 10 mm in diameter was obtained using four-step fabrication procedures: (i) interference lithography for PR grating, (ii) spin-coating of PVP polymer doped with AgNO_3_, (iii) UV laser illumination, and (iv) lift-off of PR and PVP. The Ag NP grating slab allows for photon–plasmon interaction to produce waveguide–plasmon polaritons under both the TE and TM polarizations. Continuous Ag nanoline grating was fabricated after annealing the Ag NP grating at high temperature, and exhibited Fano coupling behavior only under the TM polarization. The strong coupling between the plasmon and the waveguide modes of different orders was manipulated by altering the morphology and periodicity of the Ag grating through the concentration of the silver salt and the photoresist template, respectively. We expect that the grating–waveguide slab with strong coupling resonance offer various applications in biosensing, optical switching, and narrow-band filtering.

## Data Availability

Not applicable.
